# Evaluation of the Efficacy and Safety of Cryolipolysis in Reducing Local Adipose Tissue in Women—A Randomized Pilot Study

**DOI:** 10.1111/jocd.70149

**Published:** 2025-04-09

**Authors:** Wioleta Faruga‐Lewicka, Wiktoria Staśkiewicz‐Bartecka, Marek Kardas

**Affiliations:** ^1^ Department of Food Technology and Quality Evaluation, Faculty of Public Health in Bytom Medical University of Silesia in Katowice Zabrze Poland

**Keywords:** abdominal fat, body mass index, cryolipolysis, excessive adipose tissue, women

## Abstract

**Introduction:**

Cryolipolysis was developed in 2007 as a method of reducing excessive adipose tissue using low temperatures. During the procedure, lipid crystallization occurs at temperatures much higher than the freezing point of water in the tissues. This is followed by the recruitment of macrophages, the number of which peaks at 4 weeks and disappears at about 3 months after treatment.

**Material and Methods:**

The study involved 15 women who were divided into a control group and a study group. All subjects completed the QEB questionnaire, had body circumference measurements taken, and had a body composition analysis performed before the start of therapy and 1 month after the end of therapy. The study group underwent three cryolipolysis treatments to the abdominal area, with a treatment duration of 45 min and a temperature of −8°C. Approval from the bioethics committee was obtained prior to the study.

**Results:**

Comparing the results of the control group and the study group after 3 months of the study, differences in mean values of body fat, abdominal circumference, and BMI values are evident, while statistical analysis showed a significant difference in changes in BMI values (*p* < 0.05), while changes in body fat were on the borderline of statistical significance (*p* = 0.05).

**Conclusion:**

The study confirms that cryolipolysis is a safe method of reducing locally accumulated fat, especially in the abdominal area in women.

## 1 Introduction

Cryolipolysis was developed in 2007 as a method of reducing excessive adipose tissue using low temperatures [1]. The sensitivity of adipocytes to cold was first reported in 1902 [2], with subsequent years confirming the greater susceptibility of fat cells to low temperatures compared with surrounding tissues and their selective breakdown when exposed to cold [3]. In 2010, the Food and Drug Administration (FDA) approved the cryolipolysis procedure as a noninvasive alternative to surgery for subcutaneous fat reduction without affecting surrounding tissues [4].

During the procedure, lipid crystallization occurs at temperatures much higher than the freezing point of water in the tissues. This is followed by the recruitment of macrophages, the number of which peaks at 4 weeks and disappears at about 3 months after treatment [5]. The crystallization process results in the recognition of own cells as foreign and the initiation of inflammation in the subcutaneous adipose tissue. They are digested and removed by macrophages, and the lipids inside the adipocytes are transported to the liver and metabolized [6]. The whole process is lengthy, lasting up to several weeks after cryolipolysis [7]. Cryolipolysis is considered a safe and effective method with minimal side effects [8]. The most commonly described side effects include erythema at the treatment site, numbness, swelling, and bruising. Less common ones include pain, hyperpigmentation, and paradoxical fatty hyperplasia [9].

The aim of this study was to evaluate the effectiveness of cryolipolysis treatments in reducing locally accumulated abdominal fat in women, while monitoring changes in body composition and waist and abdominal circumference. The study aimed to determine whether the application of a series of three cryolipolysis treatments leads to a statistically significant reduction in body fat, a reduction in body circumference, and an improvement in BMI compared with a control group that did not receive the intervention. An additional aim was to assess the safety of the procedure by analyzing the incidence of side effects and possible complications after treatment.

It was hypothesized that cryolipolysis treatments lead to a significant reduction in local abdominal fat and body circumference in women, while maintaining safety and minimal risk of side effects.

## 2 Material and Methods

### 2.1 Study Design

The study was conducted between January and July 2024 at a selected professional cosmetology practice in Katowice (Silesian Voivodeship, Poland). Participants in the study were recruited on a voluntary basis after recruitment had been announced via information materials available at the cosmetology practice. Prior to taking part in the study, each person was provided with detailed information about the purpose, course, and potential benefits and risks of participating. Participants were informed that participation in the study was completely voluntary and that they could withdraw their consent to further participation at any time, without giving any reason and without any consequences. In the first stage of the study, participants completed a survey questionnaire, including questions about their health status, eating habits, and lifestyle. This was followed by an individual medical interview to exclude contraindications to cryolipolysis (e.g., cryoglobulinaemia, autoimmune diseases, and pregnancy). Prior to the treatment, participants underwent a body mass composition analysis using the bioimpedance electrical analysis (BIA) method and waist, abdominal, and hip circumference measurements. Each person underwent three cryolipolysis treatments on the abdominal area; these were carried out at −8°, the treatment time was 45 min, and treatments were performed at 6‐week intervals. One month after the third treatment, the patients reapplied for a body composition analysis and completed the questionnaire survey again.

All study participants and their parents/guardians were informed about the purpose of the study, its anonymity, and were asked to accept the rules of data sharing. Information about informed and voluntary participation in the study was at the beginning of the questionnaire. The Declaration of Helsinki of the World Medical Association guided the conduct of this study. The study was approved by the Bioethics Committee of the Medical University of Silesia in Katowice (BNW/NWN/0052/KB1/44/I/23, date of approval: 11.07.2023) in light of the Law of December 5, 1996, on the professions of physician and dentist, which includes a definition of medical experimentation.

### 2.2 Research Tools

A set of research tools was used to conduct the study, which enabled a comprehensive assessment of participants' eating habits and body composition.

#### 2.2.1 Assessment of Eating Habits

A survey questionnaire was used to conduct this part of the study, which consisted of a metric and a section created on the basis of the QEB questionnaire (Questionnaire of Eating Behavior Questionnaire for the study of eating behavior and opinions on food and nutrition) developed by the Behavioral Determinants of Food Team, PAN. This tool is widely used in epidemiological studies and allows the assessment of eating habits and opinions on food and nutrition [10].

For the QEB questionnaire, questions on respondents' dietary habits (question numbers 1–8, 11, 13, 14, 20, 21, 27–29, 34–37, 42) and frequency of food intake (question numbers 9, 10, 12, 15–19, 22–26, 30–33, 38–41) were used. For questions on the frequency of consumption of different foods, a ranking method was used, where the answer “never” received a rank of 1, “1–3 times a month” a rank of 2, “once a week” 3, “several times a week” 4, “once a day” rank 5, and “several times a day” rank 6.

#### 2.2.2 Body Composition Analysis

The second part of the study consisted of an electrical bioimpedance (BIA) body mass composition analysis using the Tanita bc 418 (Tanita Corporation, Tokyo, Japan), which is certified for medical use and meets NAWI CLASS III standards for scales used for medical measurements. The analyzer is CE0122 certified. As far as medical devices are concerned, it meets the requirements of the MDD 93/42/EEC (Medical Device Directive). Body composition assessment parameters included percentage body fat (%PB), fat‐free mass (FFM), and total body mass (BM).

The subjects' nutritional status was assessed using body mass index calculated using the formula: BMI (kg/m^2^) = body mass (kg)/height (m)^2^. Subsequently, the results were interpreted according to World Health Organization (WHO) guidelines, where a BMI value ≥ 30.00 kg/m^2^ indicates obesity, values 25.00–29.99 kg/m^2^ indicate overweight, 18.50–24.99 kg/m^2^ indicates normal body weight, values 17.00–18.49 kg/m^2^ indicate underweight, and BMI ≤ 16.99 kg/m^2^ indicates malnutrition [11].

#### 2.2.3 Fat Fold Thickness Assessment

Fat fold thickness assessments to qualify probands for the study were assessed using a Dioche brand caliper measuring 16.8 × 11 × 0.5 cm; 30 g. A minimum fold thickness of 20 mm was set.

#### 2.2.4 Cryolipolysis Treatment

The cryolipolysis procedure was performed using the Body Bell Cryo Angel 360 Small device (Body Bell, Medi‐Innova, Milan, Italy). The Cryo Angel 360 Small device complies with ISO 13485 quality management standards for medical devices and is ce 0122 certified—compliance with the MDD 93/42/EEC directive for medical devices. The device uses a 360° uniform cooling technology, which allows for a more effective and uniform effect on adipose tissue; the applicator sucks in the skin fold, which increases the efficiency of the cooling process and enables deeper penetration of the cold, leading to the selective crystallization of lipids in adipocytes.

### 2.3 Study Group

The study involved 15 women who were divided into two groups: a study group (nine subjects) and a control group (six subjects). The mean age of the women in the study was 31.4 ± 11.68. Four of them had a normal body weight (26.67%), nine were overweight (60%), and two were first‐degree obese (13.33%). The average body fat content of the entire group was 33.87%.

Inclusion criteria for the study collectively included (1) being of legal age, (2) consenting to participate in the study and to publish the results obtained, (3) having locally accumulated abdominal fat of at least 20 mm measured with a caliper, (4) completing a pre‐ and posttherapy questionnaire, and (5) having no contraindications to body composition analysis and no contraindications to cryolipolysis (i.e., pregnancy and lactation, Raynaud's syndrome, cryoglobulinaemia, cold‐induced urticaria, severe congestive dermatitis, and cold intolerance).

Exclusion criteria included (1) incorrect completion of any questionnaire, (2) nonattendance at subsequent cryolipolysis treatments.

The recruitment process used a random allocation of female participants to either the study or control group. Randomization was carried out using a computer tool, which ensured transparency and equality in the selection of participants. The control group was subjected to the same body composition measurements as the study group but did not undergo cryolipolysis treatments, allowing for the assessment of possible natural changes in body parameters unrelated to the intervention.

To eliminate confounding factors, participants were required to maintain an unchanged diet and physical activity level throughout the study. Diet was monitored using the QEB questionnaire, which participants completed both before and after the study. Physical activity, on the other hand, was monitored by the average number of steps taken per day measured with a watch, taking into account the average number of steps from the month before the start of therapy and each month of the study in turn.

### 2.4 Statistical Analysis

Statistical analysis was performed using the Statistica 13.3 software (TIBCO Software Inc., Palo Alto, CA, USA). Both parametric tests, which are used when assumptions about the normality of the data distribution are met, and nonparametric tests, which are used when these assumptions are not met, were used to analyze the data. A normal distribution was tested for conformance using the Shapiro–Wilk test. Measurable parameter values (e.g., measurement results) were presented using the arithmetic mean (average) and standard deviation.

A value of *p* < 0.05 was taken as the level of statistical significance.

## 3 Results

The frequency of consumption of each group of products by the study subjects is shown in Table 1.

**TABLE 1:** Frequency of consumption of individual product groups by subjects in the study and control groups prior to treatment therapy.

Type of product	Study group (SG)/control group (CG)	Several time a day *n* (%)	Once a day *n* (%)	Several times a week *n* (%)	Once a week *n* (%)	1–3 times a month *n* (%)	Never *n* (%)
Whole meal bread	SG (*N* = 9)	0 (0%)	0 (0%)	2 (22.22%)	2 (22.22%)	3 (33.33%)	2 (22.22%)
	CG (*N* = 6)	0 (0%)	0 (0%)	0 (0%)	3 (50%)	3 (50%)	0 (0%)
	*p*	0.67	0.67	0.53	0.39	0.52	0.53
Milk and flavored milk	SG (*N* = 9)	2 (22.22%)	0 (0%)	1 (11.11%)	1 (11.11%)	2 (22.22%)	3 (33.33%)
	CG (*N* = 6)	0 (0%)	0 (0%)	4 (66.67%)	0 (0%)	1 (16.67%)	1 (16.67%)
	*p*	0.53	0.67	0.15	0.67	0.67	0.53
Fermented milk drinks	SG (*N* = 9)	1 (11.11%)	0 (0%)	0 (0%)	2 (22.22%)	4 (44.44%)	2 (22.22%)
	CG (*N* = 6)	2 (33.33%)	0 (0%)	2 (33.33%)	2 (33.33%)	0 (0%)	0 (0%)
	*p*	0.41	0.67	0.25	0.57	0.31	0.53
Cottage cheese, homogenized cheese	SG (*N* = 9)	0 (0%)	0 (0%)	5 (55.56%)	0 (0%)	3 (33.33%)	1 (11.11%)
	CG (*N* = 6)	0 (0%)	0 (0%)	3 (50%)	0 (0%)	3 (50%)	0 (0%)
	*p*	0.67	0.67	0.63	0.67	0.52	0.67
Cheese, processed cheese	SG (*N* = 9)	0 (0%)	0 (0%)	7 (77.78%)	1 (11.11%)	0 (0%)	1 (11.11%)
	CG (*N* = 6)	0 (0%)	0 (0%)	4 (66.67%)	2 (33.33%)	0 (0%)	0 (0%)
	*p*	0.67	0.67	0.59	0.41	0.67	0.67
Meat preparations and meals	SG (*N* = 9)	0 (0%)	2 (22.22%)	7 (77.78%)	0 (0%)	0 (0%)	0 (0%)
	CG (*N* = 6)	0 (0%)	1 (16.67%)	5 (83.33%)	0 (0%)	0 (0%)	0 (0%)
	*p*	0.67	0.67	0.62	0.67	0.67	0.67
Preparations and meals of fish	SG (*N* = 9)	0 (0%)	0 (0%)	0 (0%)	3 (33.33%)	5 (55.56%)	1 (11.11%)
	CG (*N* = 6)	0 (0%)	0 (0%)	0 (0%)	2 (33.33%)	4 (66.67%)	0 (0%)
	*p*	0.67	0.67	0.67	0.69	0.58	0.67
Legume foods	SG (*N* = 9)	0 (0%)	0 (0%)	0 (0%)	1 (11.11%)	6 (66.67%)	2 (22.22%)
	CG (*N* = 6)	0 (0%)	0 (0%)	2 (33.33%)	0 (0%)	3 (50%)	1 (16.67%)
	*p*	0.67	0.67	0.25	0.67	0.55	0.67
Fried products	SG (*N* = 9)	0 (0%)	0 (0%)	6 (66.67%)	3 (33.33%)	0 (0%)	0 (0%)
	CG (*N* = 6)	0 (0%)	0 (0%)	5 (83.33%	1 (16.67%)	0 (0%)	0 (0%)
	*p*	0.67	0.67	0.55	0.53	0.67	0.67
Fast food products	SG (*N* = 9)	0 (0%)	0 (0%)	0 (0%)	1 (11.11%)	7 (77.78%)	1 (11.11%)
	CG (*N* = 6)	0 (0%)	0 (0%)	0 (0%)	5 (83.33%)	1 (16.67%)	0 (0%)
	*p*	0.67	0.67	0.67	0.09	0.19	0.67
Fruit	SG (*N* = 9)	1 (11.11%)	2 (22.22%)	6 (66.67%)	0 (0%)	0 (0%)	0 (0%)
	CG (*N* = 6)	0 (0%)	2 (33.33%	2 (33.33%)	2 (33.33%)	0 (0%)	0 (0%)
	*p*	0.67	0.57	0.40	0.25	0.67	0.67
Vegetables	SG (*N* = 9)	5 (55.56%)	1 (11.11%)	3 (33.33%)	0 (0%)	0 (0%)	0 (0%)
	CG (*N* = 6)	2 (33.33%)	1 (16.67%)	3 (50%)	0 (0%)	0 (0%)	0 (0%)
	*p*	0.49	0.67	0.52	0.67	0.67	0.67
Sweets, confectionery	SG (*N* = 9)	0 (0%)	3 (33.33%)	5 (55.56%)	0 (0%)	0 (0%)	1 (11.11%)
	CG (*N* = 6)	0 (0%)	2 (33.33)	4 (66.67%)	0 (0%)	0 (0%)	0 (0%)
	*p*	0.67	0.69	0.58	0.67	0.67	0.67
Carbonated drinks	SG (*N* = 9)	0 (0%)	0 (0%)	1 (11.11%)	3 (33.33%)	2 (22.22%)	3 (33.33%)
	CG (*N* = 6)	0 (0%)	0 (0%)	0 (0%)	3 (50%)	3 (50%)	0 (0%)
	*p*	0.67	0.67	0.67	0.52	0.40	0.41
Energy drinks	SG (*N* = 9)	0 (0%)	0 (0%)	0 (0%)	1 (11.11%)	2 (22.22%)	6 (66.67%)
	CG (*N* = 6)	0 (0%)	0 (0%)	0 (0%)	0 (0%)	1 (16.67%)	5 (83.33%)
	*p*	0.67	0.67	0.67	0.67	0.67	0.55
Alcohol	SG (*N* = 9)	0 (0%)	0 (0%)	1 (11.11%)	1 (11.11%)	4 (44.44%)	3 (33.33%)
	CG (*N* = 6)	0 (0%)	0 (0%)	1 (16.67%)	1 (16.67%)	3 (50%)	1 (16.67%)
	*p*	0.67	0.67	0.67	0.67	0.63	0.53

Abbreviations: CG, control group; SG, study group.

During the course of therapy, body circumference measurements were taken after each treatment and a body composition analysis was performed. Detailed results are shown in Table 2.

**TABLE 2:** Changes in body circumference and body fat content during treatment therapy.

Patient	Before treatment	After 1 treatment	After 2 treatments	After 3 treatments	Difference	*p*
**Changes in body fat content during therapy (%)**						
1	37.74%	36.30%	36.12%	37.33%	−0.41%	*p* = 0.31
2	33.23%	33.38%	32.24%	34.02%	0.79%	
3	33.08%	28.86%	31.28%	28.67%	−4.41%	
4	35.00%	33.44%	34.57%	34.53%	−0.47%	
5	40.21%	38.86%	39.24%	39.24%	−0.97%	
6	29.02%	29.40%	27.89%	27.77%	−1.25%	
7	41.74%	37.38%	38.58%	36.13%	−5.61%	
8	29.46%	32.05%	33.20%	33.11%	3.65%	
9	34.58%	34.02%	34.98%	34.44%	−0.14%	
**Changes in abdominal circumference during therapy (cm)**						
1	83.00	82.00	83.00	86.00	3.00	*p* = 0.32
2	85.00	82.00	82.00	84.00	−1.00	
3	91.50	91.50	93.00	92.00	0.50	
4	91.00	88.00	86.00	86.00	−5.00	
5	90.00	90.00	90.00	90.00	0.00	
6	81.00	79.00	77.00	78.00	−3.00	
7	116.00	113.00	111.00	111.00	−5.00	
8	79.00	77.00	74.00	82.00	3.00	
9	94.00	90.00	92.00	92.00	−2.00	
**Changes in BMI during therapy**						
1	26.85	26.16	26.16	26.51	−0.34	*p* = 0.19
2	22.91	22.48	22.64	22.64	−0.27	
3	29.11	28.78	28.85	28.70	−0.41	
4	28.13	27.09	26.66	26.49	−1.64	
5	30.21	29.80	29.90	29.90	−0.31	
6	23.71	23.38	23.42	23.67	−0.04	
7	34.58	32.27	31.89	31.04	−3.54	
8	25.80	25.68	26.60	26.52	0.72	
9	27.87	28.11	27.87	28.28	0.41	

The detailed results of the control group are shown in Table 3.

**TABLE 3:** Results of the control group.

Patient	Before	After 3 months	Difference	*p*
**Changes in body fat over time (%)**				
K1	30.20%	31.10%	0.90%	*p* = 0.16
K2	36.70%	39.20%	2.50%	
K3	28.36%	29.11%	0.75%	
K4	33.56%	32.50%	−1.06%	
K5	29.67%	30.11%	0.44%	
K6	35.55%	36.67%	1.12%	
**Changes in abdominal circumference over time (cm)**				
K1	86	84	−2	*p* = 0.18
K2	93	101	8	
K3	79	82	3	
K4	93	96	3	
K5	88	88	0	
K6	93	94	1	
**Changes in BMI over time**				
K1	22	22.35	0.35	*p* = 0.02*
K2	26.3	27.6	1.3	
K3	26.67	27.33	0.66	
K4	28.56	28.76	0.2	
K5	22	22.38	0.38	
K6	26.1	27.45	1.35	

*Note:* *p < 0.05.

A comparison of the results from the study group and the control group is shown in Table 4.

**TABLE 4:** Comparison of results from the study group and the control group.

	Before (*X* ± SD)	After (*X* ± SD)	*p*
**Changes in body fat content (%)**			
Study group	34.90 ± 0.04	33.92 ± 0.03	*p* = 0.05
Control group	32.34 ± 0.03	33.12 ± 0.04	
**Changes in abdominal circumference (cm)**			
Study group	90.06 ± 10.38	89 ± 8.91	*p* = 0.21
Control group	88.67 ± 5.12	90.83 ± 6.74	
**Changes in BMI**			
Study group	27.69 ± 3.32	27.08 ± 2.58	*p* = 0.001*
Control group	25.27 ± 2.45	25.98 ± 2.60	

Abbreviations: SD, standard deviation; X, average.

* *p* < 0.05.

Comparing the results of the control group and the study group after 3 months of the study, differences in mean values of body fat, abdominal circumference and BMI values are evident, while statistical analysis showed a significant difference in changes in BMI values (*p* < 0.05), while changes in body fat were on the borderline of statistical significance (*p* = 0.05).

## 4 Discussion

Cryolipolysis is described as a safe, noninvasive method of reducing excessive, locally accumulated adipose tissue. Side effects of cryolipolysis include paradoxical adipose tissue hypertrophy (PAH). A multicentre study involving 8658 treatment cycles in 2114 patients reported an incidence of PAH of between 0.05% and 0.39% [12]. Some studies indicate a higher incidence trend of PAH in men, Hispanics, and in the abdominal region [3, 12, 13].

Posttreatment complications such as severe frostbite [14], cold burns [15], cold panniculitis [16] or submental consideration [17, 18] can also be found in the literature. Among the most frequently mentioned postoperative sensations, erythema, bruising, and paresthesias are indicated [19, 20]. On the other hand, the incidence of side effects can be influenced by a large number of factors, including the quality of the device, the duration of the procedure, and a thorough patient interview. There is a lack of available data on what treatment parameters allow the therapy to be effective and safe. The recommended duration of one session and the target treatment temperature also vary considerably depending on the manufacturer of the equipment. In our study, a duration of one treatment session of 45 min and a temperature of −8°C were used. The chosen treatment parameters were characterized by both the effectiveness and safety of the overall therapy, as none of the patients experienced the previously mentioned side effects.

The own study is innovative among the available literature data due to the body composition analysis carried out before the start of the treatment therapy and 1 month after the last treatment of the series, which allows for a real assessment of the treatment's effectiveness. The mean reduction in patients' body fat was 0.98% (before treatment 34.90 ± 0.04, after treatment 33.92 ± 0.03), the mean reduction in abdominal circumference was 1.06 cm (before treatment 90.06 ± 10.38, after treatment 89 ± 8.91), while the change in BMI was reduced by an average of 0.60 (before treatment 27.69 ± 3.32, after treatment 27.08 ± 2.58).

A review of studies by Hetzel et al. [21] showed that cryolipolysis treatment is also an effective method of fat reduction. The average reduction in fat tissue thickness in the studies analyzed ranged from 2 to 5.1 mm after 1–3 treatments.

A study by Vignoli and Marmol [22] on a group of 287 patients investigated the efficacy and safety of cryolipolysis treatment. The number of treatments and body parts varied according to patient preference and treatment indications. An average fold thickness reduction of 24.64 ± 7.14 mm was achieved.

A similar topic was undertaken by Lopes‐Martins et al. [23]. Thirty overweight and obese women were enrolled in the study and consecutively underwent three cryolipolysis treatments each. An average reduction in body fat of 4.1 kg and a reduction in BMI of 0.7 points were achieved. The researchers also measured the patients' cholesterol levels before and after treatment. Analysis showed a reduction in total cholesterol by an average of 15.7 mg/dL and a reduction in LDL fraction cholesterol by 10.2 mg/dL.

A study by Bellocco et al. [24] evaluated the effectiveness of cryolipolysis treatment in reducing abdominal fat in 15 women. They were divided into three groups based on the temperature used during the procedure (Group I: −2°C, Group II: −3°C, Group III: −4°C). Three treatments were performed on each participant. A reduction in plicometric measurements was observed in groups II and III. A greater tendency to redness after treatment was also noted in group III.

Choi et al. [25] conducted a study on a group of 25 patients for the abdominal region. They assessed the thickness of adipose tissue using calipers both before therapy and 12 weeks after the first treatment. The mean thickness of subcutaneous abdominal fat in the analysis after 12 weeks was lower (40.4 ± 6.8 mm) compared to before treatment therapy (49.3 ± 8.5 mm). Differences were also seen in abdominal circumference, with a reduction of 1.02 ± 0.41 cm at 12 weeks after the first treatment.

The data presented indicate that the treatment is moderately effective in fat reduction and that the safety of the therapy is high. Reliable studies are still lacking to draw long‐term conclusions and develop increasingly effective treatment protocols.

### 4.1 Strengths and Weaknesses of the Study

The strengths of the study are that the probands had a body composition analysis before and after the therapy, controlling for body fat, which is the main focus of the cryolipolysis treatment. Assessment of body fat was performed 1 month after the last treatment in order to realistically assess the effectiveness of the therapy. The test group received three treatments each, which is the number recommended by device manufacturers for this type of treatment. Treatments were performed on the same area for each proband.

Limitations of the study include several important issues. First, the small size of the study group (15 participants) and the restriction of the study to women only may affect the generalizability of the results to a wider population. Additionally, the influence of other factors, such as lifestyle, could have potentially affected the results, even though the participants were required to maintain an unchanged lifestyle. Finally, the study was limited to assessing the efficacy of a single series of treatments using specific parameters, which may not reflect the full range of possibilities of this method in different treatment configurations.

It would also be beneficial to expand testing to include biochemical measurements such as glucose levels and liver parameters before and after the start of treatment therapy.

## 5 Conclusion

The study confirms that cryolipolysis is a safe method of reducing locally accumulated fat, especially in the abdominal area in women. Although the results indicate a moderate efficacy of the therapy, the mean body fat was reduced by 0.98% and the mean abdominal circumference decreased by 1.06 cm. In addition, a decrease in BMI of 0.60 was observed, highlighting the potential of this method as a tool to help improve body shape.

An important aspect is the absence of significant side effects, confirming the high safety of this technique.

The results of the study have important practical implications for the cosmetology and aesthetic medicine industry. Cryolipolysis, as a safe and moderately effective method for the reduction of local adipose tissue, can be recommended to those looking for noninvasive ways to improve the silhouette, especially in the abdominal area. Carrying out a series of three treatments at appropriate intervals (6 weeks) and monitoring body composition can increase the effectiveness of the therapy and enable better control of results.

## Author Contributions

W.F.‐L.: methodology, conceptualization, project administration, funding acquisition, writing – original draft. W.S.‐B.: conceptualization, project administration, methodology, formal analysis. M.K.: formal analysis, software, writing – review and editing, visualization, investigation, supervision.

## Ethics Statement

The study was approved by the Bioethics Committee of the Silesian Medical University in Katowice (BNW/NWN/0052/KB1/44/I/23. date of approval: 11.07.2023) in light of the Act of December 5, 1996, on the professions of physician and dentist, which includes the definition of medical experiment.

## Conflicts of Interest

The authors declare no conflicts of interest.

## Data Availability

The data that support the findings of this study are available from the corresponding author upon reasonable request.
